# SKINOMICS: Transcriptional Profiling in Dermatology and Skin Biology

**DOI:** 10.2174/138920212801619241

**Published:** 2012-08

**Authors:** Miroslav Blumenberg

**Affiliations:** The Departments of Dermatology, Biochemistry and Molecular Pharmacology, and the NYU Cancer Institute, NYU Langone Medical Center, New York, NY, USA

**Keywords:** Bioinformatics, disease markers, epidermal differentiation, melanoma, microarrays, psoriasis, UV damage.

## Abstract

Recent years witnessed the birth of bioinformatics technologies, which greatly advanced biological research. These ‘omics’ technologies address comprehensively the entire genome, transcriptome, proteome, microbiome etc. A large impetus in development of bioinformatics was the introduction of DNA microarrays for transcriptional profiling. Because of its accessibility, skin was among the first organs analyzed using DNA microarrays, and dermatology among the first medical disciplines to embrace the approach. Here, DNA microarray methodologies and their application in dermatology and skin biology are reviewed. The most studied disease has been, unsurprisingly, melanoma; markers of melanoma progression, metastatic potential and even melanoma markers in blood have been detected. The basal and squamous cell carcinomas have also been intensely studied. Psoriasis has been comprehensively explored using DNA microarrays, transcriptional changes correlated with genomic markers and several signaling pathways important in psoriasis have been identified. Atopic dermatitis, wound healing, keloids etc. have been analyzed using microarrays. Noninvasive skin sampling for microarray studies has been developed. Simultaneously, epidermal keratinocytes have been the subject of many skin biology studies because they respond to a rich variety of inflammatory and immunomodulating cytokines, hormones, vitamins, UV light, toxins and physical injury. The transcriptional changes occurring during epidermal differentiation and cornification have been identified and characterized. Recent studies identified the genes specifically expressed in human epidermal stem cells. As dermatology advances toward personalized medicine, microarrays and related ‘omics’ techniques will be directly applicable to the personalized dermatology practice of the future.

## INTRODUCTION

‘SKINOMICS’ is a field of Bioinformatics applied specifically to dermatology and skin biology. Bioinformatics refers to a new set of technologies that greatly advance our capabilities for research by acquiring, managing, and processing biological information, including medical, genetic, biochemical, and biophysical data [1]. Bioinformatics deals with large volumes of data and involves computer-based resources such as databases, algorithms, computational and statistical techniques, as well as new theoretical approaches to deal with the issues arising from the sheer size of the data [2, 3]. Bioinformatics is defined by its methodology rather than the object of study (akin to, e.g.; microscopy). The ‘omics’ studies address comprehensively the entire genome, transcriptome, proteome, microbiome etc.; producing previously unimaginable volumes of raw data.

Bioinformatics comprises four major types of activities: 1. data acquisition, 2. database development, 3. data analysis, 4. integration and analysis of the integrated data. The analysis algorithms are usually custom-designed for a specific application and have become a wholly new field of creative research that bridges, sometimes uneasily, biology and computer sciences. A key task in bioinformatics is to represent visually the integrated data, to facilitate better understanding of the underlying biological complexities. The overarching goal of omics approach is termed “systems biology”, because it encompasses a broad and comprehensive view of its topic, an integrated biological “system.” [4].

However, for the promises of bioinformatics to be fulfilled we need new, sophisticated approaches that can make adequate use of the massive amounts of data available. It has become impossible for individual researchers to integrate massive amounts of data into their research programs. Analysis of complex systems that comprise data from several sources requires collaborations sharing data across institutions and increasingly, important discoveries are made by teams of scientists who combine different skill sets, biologists, computer scientists, statisticians and data-visualization experts. This data-rich approach to science is challenging because the speed of the internet has not kept pace with the growth of scientific data sets. Working with large data sets, it is often easier to send computations to the data, rather than download the data to one’s workstation.

## DNA MICROARRAYS

A large momentum in bioinformatics was created by the development of DNA microarrays for transcriptional profiling [5]. Microarrays follow a simple concept: they combine dot blots and northern blots, but reverse the hybridization – the probes are put on a solid support and the label on the bulk RNA. A microarray is a small piece of solid support, a glass slide, nylon membrane, silicon chips etc.; onto which DNA sequences from thousands of different genes are attached at fixed locations. The DNA is spotted, printed, or directly synthesized on the support. There are several important attributes of DNA microarrays: 1. they probe lots of genes, e.g.; all human genes, creating massively parallel experiments; 2. they are tiny, so they require only minute amounts of RNA; 3. they measure RNA quantitatively (almost), which allows making comparisons easy, for example comparing cancerous *vs*. healthy tissue, treated *vs*. untreated etc.

There are two basic types of DNA microarray technologies: cDNA and oligonucleotide. cDNA microarrays are usually produced ‘in-house’, in core facilities, using robotics. The cDNAs are usually PCR-amplified segments of plasmids; this means that spotting microarrays requires a large and expensive quality-controlled library of plasmids. The spotting uses ordinary glass slides, which makes the 2nd microarray very cheap, less than $10. However, the spotting of the first microarray requires robotics, a validated library and many quality controls; it can easily cost more than a million.

For cDNA microarrays, one labels two RNA samples, e.g.; healthy *vs*. diseased, with fluorescent dyes of different colors, usually red and green. The two labeled RNAs are mixed and hybridized to the microarray, the microarray washed and scanned using red and green wavelengths separately. This allows one to calculate the ratios of the two RNAs hybridized to a given spot, i.e.; relative ratios of RNA amounts in the healthy *vs*. diseased sample. A skin-specific cDNA array, named DermArray, containing >4000 gene probes is commercially available [6].

Alternative to cDNA, the oligonucleotide microarrays are commercially available from companies including Affymetrix, Illumina, Agilent, etc. A single RNA sample is hybridized to each microarray, which means 2 microarrays are necessary for a comparison. But, one can simultaneously compare many different samples, not just get a ratio of 2 samples. This is ideal when comparing many patient biopsies in a cohort, a time course of treatment etc. An oligonucleotide microarray is significantly more expensive, $500 or more, but, the suppliers provide extensive quality control and the software for analysis.

Affymetrix microarray unit the array of some 200,000 hybridization spots Fig. (**1a**). The hybridization oven Fig. (**1b**) can hold many microarrays, over 60; the hybridization takes usually overnight. The ‘fluidics’ station Fig. (**1c**) processes the microarrays after hybridization: adds dyes and antibodies, washes the microarrays through several cycles etc. This operation takes approximately 2 hrs. The scanner Fig. (**1d**) works fast, 7 min/microarray, and deposits the data into a computer. The entire set-up fits atop a regular lab bench Fig. (**1e**).

Affymetrix microarrays contain essentially all human genes. In Fig. (**2**) the hybridization intensity of an entire microarray is represented and, as we zoom in, we see small squares of hybridization; intense bright signal represents genes expressed at high level, and dark squares genes that are not expressed. Each human gene is probed with at least 11 different DNA sequences, making 11 measurements for each gene. This redundancy provides statistically significant results.

There are five phases in a microarray analysis: 1. Prepare (or buy) the DNA microarray with the chosen target DNAs. 2. Purify RNA from the sample and label it. 3. Hybridize the labeled samples to the DNA microarray. 4. Detect and measure the bound RNA/DNA, and save data in a computer (data acquisition). 5. Lastly, analyze the data using computational methods.

There are several steps in data acquisition (phase 4): The first is to scan the arrays, quantitate each spot, subtract background, normalize all microarrays in a series to the same overall intensity, and finally, export a table of results. This table contains the levels of RNA expression for all genes. Transcription profiling produces very large data sets, and therefore analyses become more complex, and data increasingly difficult to document and reproduce.

Microarray data are deposited into a few large, quality-controlled, annotated and curated databases established by the biomedical research community. Currently there are more than a thousand molecular biology databases, the largest being The National Center for Biotechnology Information (NCBI) Gene Expression Omnibus, GEO (http:// ncbi.nlm.nih.gov/geo) [7]. GEO contains more than 330,000 gene expression profiles and has annual growth rate of 150%! There are also smaller, disease-specific repositories of microarray data. Submitting data in these repositories has become a condition for publication in most journals nowadays. Often data are loaded into databases even before the analysis is complete.

Diverse integration approaches for molecular biological data sources have been developed to combine and present heterogeneous microarray data. The majority of tools were developed as academic freeware distributed on the Internet, although commercial companies also provide proprietary databases, algorithms and tools. Meta-analysis approaches have been developed to integrate multiple microarray studies [8]. For example, the OncoMINE database uses a large collection of microarray data to identify genes differentially expressed in cancer or among different cancer types (https://www.oncomine.org/resource/login.html) [9]. Dr Noh and his group were among the first to use metaanalysis of microarray data in dermatology [10].

Importantly, all steps in a microarray experiment can be fully automated, i.e.; performed automatically by robotics and software. This means that the results can be reproducible and consistent. Even artifacts in the image can be automatically recognized by the software and corrected, etc. Affymetrix’ and other automated systems can autonomously compute expression values for the 30,000 human genes and directly upload these into a database for analysis.

Unfortunately, the outputs of the microarray results are usually very large data tables; these are rather difficult to interpret and require expertise to analyze. Data are often presented as simplified multicolored graphs, which are still not easy to interpret and can be actually confusing and ambiguous. The simple and intuitive but meaningful representation of the microarray results is currently the biggest stumbling block to their more widespread use.

## MICROARRAYS IN DERMATOLOGY AND SKIN BIOLOGY

Because of its accessibility, skin has been among the first organs analyzed using DNA microarrays [11]. The first microarrays were developed at Stanford University by Pat Brown and his group in 1995 [5]. Soon thereafter, in 1999, microarrays were used in skin biology to show that dermal fibroblasts respond to serum by inducing the wound healing responses [11]. Dr. Paul Khavari with his group was the first to use microarrays applied to a problem in dermatology [12, 13]. They used microarrays to study the effects of replacement gene therapy for junctional epidermolysis bullosa, a lethal genetic disorder caused by mutations in laminin genes. Replacement of the affected gene or protein restored normal growth and adhesion to JEB cells; however, gene expression profiling showed that the gene expression has not been fully normalized. Since these pioneering studies, melanomas, basal and squamous cell carcinomas have been intensely investigated as well as psoriasis, one of the most common human inflammatory diseases, keloid formation, wound healing etc.

Specifically in dermatology, it is very important that the sample acquisition from skin can be noninvasive. Work by Dr Benson demonstrated that tape stripping can provide material of adequate quality and quantity for use in microarrays. The usefulness of this methodology has been demonstrated in psoriasis and in melanoma [14, 15].

Epidermal keratinocytes have been the target of many studies because they respond to a rich variety of inflammatory and immunomodulating cytokines, hormones, vitamins, UV light, toxins and physical injury [16-20]. Transcriptional mechanisms that regulate epidermal differentiation and cornification have begun to yield their mysteries, and very exciting recent studies identified the genes specifically expressed in epidermal stem cells [21]. Thus, skin has everything: stem cells, differentiation, signaling, inflammation, diseases, cancer, etc.; all these exciting facets of skin have been explored using DNA microarrays.

## MELANOMA AND MELANOCYTES

Arguably, the most advanced and most important microarray studies in dermatology concern melanomas. Melanoma is among the most aggressive human cancers and it is not surprising that melanoma was one of the earliest targets of DNA microarray studies: one year after the first cDNA arrays were described [22]. Microarrays have been used in comparison of metastatic and non-metastatic melanomas [23], in the integration of transcriptional and genetic data in melanoma [24], and in the development of non-invasive sample harvesting from melanomas and pigmented lesions [15]. The natural progression of melanoma includes a multi-step pigmented nevus to melanoma transition. Several groups compared the transcriptomes of pigmented moles, primary melanomas and melanoma metastases [25-27]. The molecular mechanisms of melanoma progression were examined by comparative transcriptional profiling of melanoma metastases and melanoma cell lines *vs*. normal human melanocytes [28]. Laser capture microscopy was used to dissect the tumor cells from their surroundings [29]. This study identified classes of genes that accurately discriminate normal skin, nevi, primary melanomas, and the two types of metastatic melanomas. Interestingly, the metastatic samples exhibited two distinct patterns of gene expression, similar either to flat or to nodular components of large primary melanomas. The studies that compared malignant melanomas with benign nevi identified several hundred differentially expressed novel, specific markers of the malignancy [28].

The ‘next generation’ DNA sequencing technologies are revolutionizing many areas of genomics [30] especially in genome wide mutation discovery [31, 32]. As the costs of DNA sequencing decline, this methodology may eventually supplant the microarray-based one. In skin biology and dermatology, DNA sequencing has been used primarily in melanoma research. Specifically, using deep sequencing it was shown that sun-exposed human skin contains mutations in the p53 tumor suppressor gene in up to 14% of all epidermal cells [33]. Sequencing was applied as a sensitive and cost-effective method for BRAF genotyping of melanomas from patients [34]. Massively parallel sequencing of RNA was used to define the set of microRNAs, in human melanomas [35]. Samples from melanoblasts, melanocytes, congenital nevi, and acral, mucosal, cutaneous and uveal melanomas revealed a total of 539 known microRNAs, along with the prediction of 279 novel microRNA candidates [35]. Some of the novel microRNAs may be specific to the melanocytic lineage and could be used as biomarkers in detection of metastases.

## PSORIASIS AND INFLAMMATION

DNA microarrays have been used to define the transcriptional responses of epidermal keratinocytes to various agents important in dermatology, such as: UV light, corticosteroids, retinoids etc. [16, 17, 20, 36] Our team focused on the transcriptional effects of proinflammatory and immunomodulatory cytokines and growth factors, such as IFNγ, TNFα, IL-1, OncostatinM M, TGFβ, IL-12, etc. [19, 37-40]. Specifically, for each experiment we grew up a single large batch of keratinocytes, starved them for 24h in minimal, unsupplemented medium, and then treated them with the mentioned agents. We harvested cells 1, 4, 24 and 48 hrs after the treatment and, in all cases, simultaneously harvested a treated and an untreated culture Fig. (**3**). This way, direct comparisons due specifically to the treatment, and not e.g., to culture growth, age or level of differentiation can be made. The main strength of the analysis is the identification of global changes in the biological processes, molecular functions, signaling networks or disease-associated genes. The primary weakness of microarray analysis is that differential expression of individual genes is not always reliable, and should be confirmed independently.

In collaboration with Dr Krueger and his group, we compared psoriasis with other proliferative skin diseases [41]. Dr Krueger and collaborators, leaders in the use of microarrays in psoriasis, studied the activation of T cells, recognized the importance of the IL-17 network, connected transcriptional and genetic data and demonstrated aberrant gene expression even in the resolved psoriatic lesions [42, 43]. In a tour-de-force study, the group of Gudjonsson analyzed 180 biopsy samples of healthy, lesional and non-lesional psoriasis patients, providing a large trove of data for analysis [44].

## STUDIES FOCUSED ON UV LIGHT

Several research laboratories have analyzed the transcriptional effects of UV light in epidermal keratinocytes using microarrays [20, 45, 46]. These studies were followed up by studies of UV-irradiated skin of human volunteers [47, 48]. The lists of UV-regulated genes in all studies were remarkably congruent, almost identical, especially bearing in mind the considerable differences in experimental approaches, countries of origin and the time-frames when the experiments were performed. Keratinocytes respond to UV by inducing cell repair programs, but also act to protect the underlying organism. Interestingly, like in the parable of the blind men and the elephant, while starting from very similar data, different researchers emphasized different aspects of the results: our group focused on the metabolic effects and epidermal differentiation, Sesto *et al*. on DNA repair, Murakami *et al*. on oncogenes, while Howell *et al*. focused on angiogenesis [20, 45, 46]. The large volume of data provided by microarrays allows researchers to demonstrate individual preferences, interests and inclinations. In keratinocytes exposed to γ-irradiation or X-rays, the transcriptional changes were similar to those in the UV-treated cells; specifically, the genes involved in energy metabolism were induced [49, 50].

## SKINOMICS IN OUR FUTURE

Clinical use of bioinformatics at the bedside will soon be commonplace. Microarrays will be used for disease classification and sub-classification, severity assessment and prognosis. They will be used to suggest treatments (for example to determine that a particular patient will be more responsive to drug A than to drug B), to monitor treatment efficacy and for early detection. Diseases such as Alzheimer’s and post-traumatic stress disorder have characteristic expression profile “signatures” in blood, detectable using microarrays [51, 52]. Notably, melanoma also has a characteristic signature in blood due to reduced interferon signaling (which is not necessarily melanoma-specific, however) [53].

Dermatology is in position to exploit the power of microarrays quickly and efficiently. DNA microarrays offer tremendous potential for enhanced understanding of the healthy and pathological processes in skin, including neoplasms, inflammatory diseases, genodermatoses, wound healing, cosmetic dermatology etc. The increased understanding will lead to identification of targets for treatment and, hopefully, more effective drugs with fewer side effects. Development of bed-side uses of bioinformatics methods and the concomitant price reduction of the materials and methods holds great promise for improved diagnosis, treatment and prevention of dermatologic disorders. We expect that the first personalized approaches will appear in dermatology practice before long. Soon afterwards they will become a fully accepted, standard methodology.

All kinds of skin lesions can be analyzed using microarrays. Carcinomas and melanomas will be typed and sub-typed and the prognosis and treatment will be suggested. Using similar types of microarrays, skin microbiome can be comprehensively characterized and harmful bacteria and fungi identified [54-56]. Human papilloma virus, if present, can be easily genotyped [57]. We cannot even imagine many future developments involving the use of microarrays in dermatology.

The microarray technology exists today, but its costs are too high, and it is not yet user-friendly. It could use some improvement in the laboratory methods, but it needs much more improvement in the data analysis and in the presentation and interaction software. It needs the means to convert impenetrable data that only a computer specialist can love, into forms that can be useful to dermatologists in the clinical setting.

The increased knowledge and understanding will lead not only to improved therapies for skin diseases, but also to capability to enhance the function of healthy skin. It will lead to improvements in disease prevention and, important in dermatology, to cosmetic advances as well. If all this sounds Pollyanna-ish, note that the DNA microarray methodology is only about a dozen years old and that its achievements will most likely surpass even the most optimistic predictions.

## Figures and Tables

**Fig. (1):**
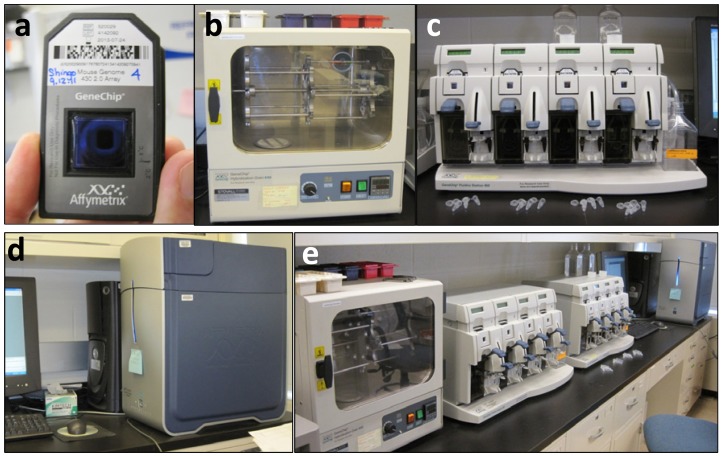
Affymetrix DNA microarrays lab ware. **A**) Microarray. **B**) Hybridization oven. **C**) Fluidics station. **D**) Scanner. **E**) Entire setup on a lab bench.

**Fig. (2):**
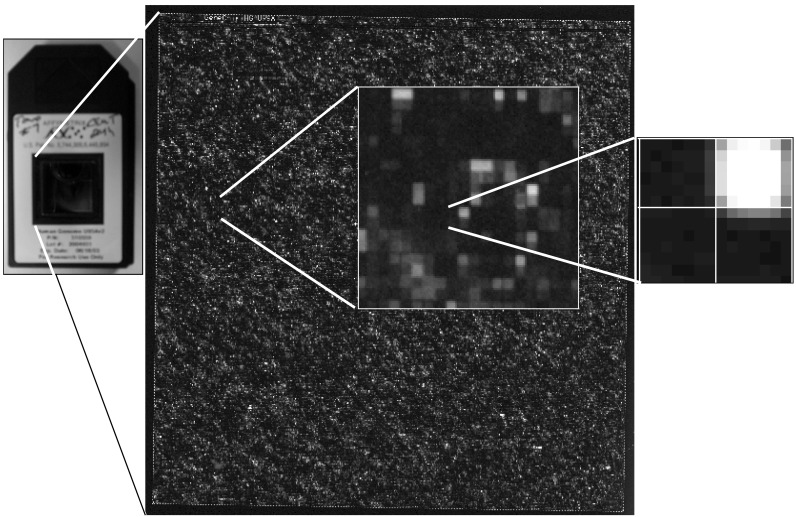
Image of a DNA microarray hybridization, with zoom-in to individual pixels.

**Fig. (3):**
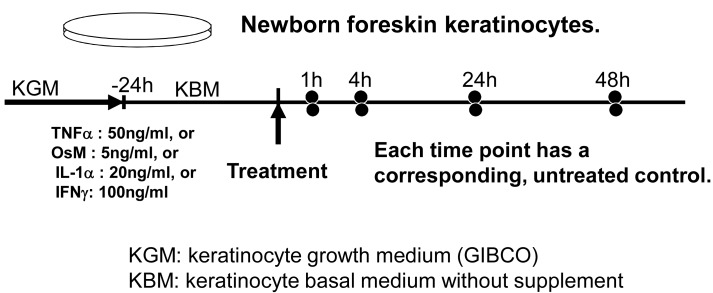
Our experimental protocol. Uniform approach allows cross-comparisons among the experiments.
